# Anti-Inflammatory Activity of Glyceryl 1,3-Distearate Identified from *Clinacanthus nutans* Extract against Bovine Mastitis Pathogens

**DOI:** 10.3390/antibiotics12030549

**Published:** 2023-03-09

**Authors:** Saruda Thongyim, Salinee Chiangchin, Hataichanok Pandith, Yingmanee Tragoolpua, Siriphorn Jangsutthivorawat, Aussara Panya

**Affiliations:** 1 Doctor of Philosophy Program in Biology (International Program), Faculty of Science, Chiang Mai University, Chiang Mai 50200, Thailand; 2 Department of Biology, Faculty of Science, Chiang Mai University, Chiang Mai 50200, Thailand; 3 Research Center in Bioresources for Agriculture, Industry and Medicine, Faculty of Science, Chiang Mai University, Chiang Mai 50200, Thailand

**Keywords:** anti-inflammatory activity, bovine mastitis, *Clinacanthus nutans*, LPS-induced cell death, plant extracts

## Abstract

*Clinacanthus nutans* is widely used as a traditional medicine in Thailand and other countries in Southeast Asia. Although its effectiveness is well documented, its therapeutic use is limited to the treatment of only a few diseases; mostly it is used as an anti-viral agent against varicella-zoster and herpes simplex virus infections. Herein, we demonstrate the therapeutic activity of *C. nutans* extracts in lowering inflammation in a model of bovine mastitis caused by bacterial infection. Lipopolysaccharide (LPS), a gram-negative bacterial component, caused inflammation activation in bovine endothelial cells (CPAE) through the upregulation of proinflammatory cytokines (*IL6* and *IL1β*) and chemokines (*CXCL3* and *CXCL8*) gene expression, partially leading to cell death. Treatment with *C. nutans* crude extract significantly diminished these responses in a dose-dependent manner. The solvent fractionation of *C. nutans* extract revealed that the ethyl acetate (C_4_H_8_O_2_) fractions had a high potential to protect against cell death and diminished *IL1β*, *IL6*, *CXCL3*, and *CXCL8* levels to less than 0.45 folds relative to the LPS-treated control. Glyceryl 1,3-distearate (C_39_H_76_O_5_) was identified as a bioactive compound responsible for the anti-inflammation activity but not the anti-cell death activity of *C. nutans* extract. This study highlighted the efficiency of *C. nutans extracts* as an alternative therapeutic option for the natural-product sustainable development of bovine mastitis treatment.

## 1. Introduction

*Clinacanthus nutans* (Burm.f.) Lindau, known as Phaya Yo or Saled Pangpon Tua Mea in Thai, is included in Thailand’s National List of Essential Herbal Medicines (2016) [1]. It belongs to the Acanthaceae family which is widely distributed in Southeast Asian countries as well as in China [2,3]. The parts of this plant mainly the leaves have been used traditionally to treat several virus infectious diseases and injuries, such as burns, skin rashes, and animal bites [2,4]. To date, the pharmacological activities of *C. nutans* have been reported, revealing the broad-range biological activities of *C. nutans* extract, which can be applied for therapeutic uses owing to its anti-virus [5,6], anti-inflammatory [7], anti-bacterial [8,9], anti-tumorigenic [10], and immunostimulating activities [7,10]. Unfortunately, only a few *C. nutans* products are commercially available currently and are mainly limited to the treatment of varicella-zoster and herpes simplex virus infections. To support and encourage the sustainable development and conservation of *C. nutans* for therapeutic use; therefore, additional studies to elucidate the therapeutic benefits of *C. nutans* against various disease models are needed.

Bovine mastitis is an important disease that economically impacts the dairy cattle industry worldwide [11]. It was found to be present in approximately 57.1% of cows, with the proportion of subclinical mastitis (SCM) being determined at 19.0%. Considering only this SCM proportion, the economic loss was reported to exceed USD 1 billion annually for the United States dairy industry by decreasing milk production and quality [11]. *Escherichia coli* was reported to cause acute mastitis resulting in udder health and milk quality impairment, not only during the clinical phase but also in the long term after healing [12]. Infection with *E. coli* can trigger acute inflammation as a host response to pathogenic microorganism attack and possibly lead to serious complications. Inflammation was clearly found to occur in affected glands with mild to severe symptoms [13]. Lipopolysaccharide (LPS) is a glycolipid in the gram-negative bacterial cell wall and functions as a bacterial toxin. This large molecule is comprised of lipid A (hydrophobic domain), a core oligosaccharide, and a distal polysaccharide (O antigen) which is linked with covalent bonds [14]. LPS is recognized as an endotoxin of gram-negative bacteria that can promote a strong immune response and eventually promote acute inflammation via massive cytokine release, mainly from immune cells. LPS binds to LPS binding protein (LBP) upon entering the blood circulation. LPS is then transferred to the CD14 protein, which presents LPS to the Toll-like receptor 4 (TLR4) and MD-2 (TLR4-MD-2) receptor complexes on host cells. These interactions activate the signaling cascade of NF-κB, resulting in the production of pro-inflammatory cytokines, i.e., interleukin 1β (IL1β), interleukin 6 (IL6), interleukin 8 (IL8/CXCL8), and tumor necrosis factor α (TNF-α), in addition to triggering the infiltration of polymorphonuclear cells (neutrophils, PMNs) to the site of infection [15]. Additionally, the upregulation of chemokine ligand 3 (*CXCL3*) and chemokine ligand 8 (*CXCL8*) upon LPS stimulation are the main factors in the induction of the migration of neutrophils toward the site of inflammation, which could amplify the inflammation response magnitude [16]. Consequently, this inflammation can cause the reduction of milk yield and quality, increasing the need for culling of affected cows, and death in dairy cattle. Neutralization of the inflammation response is therefore needed to prevent complications and lower the cost of therapeutic management. The most common treatment method available against bovine mastitis is the administration of antibiotic drugs, i.e., streptomycin, ampicillin, cloxacillin, penicillin, and tetracycline [17]. However, their uses are associated with the problem of anti-microbial resistance, which points to the requirement for alternative anti-microbial agents or resistance modifiers to combat the widespread phenomenon of antibiotic resistance.

Natural substances have been attracting great attention in developing alternative approaches for treating diseases due to their diverse biological activities and high safety. Since plant-based medicine naturally promotes synergistic effects, it represents high potential for supplying agents that can obviate multidrug resistance mechanisms. Recently, our research group reported the feasible activity of *C. nutans* extract in controlling bacterial growth in a bovine mastitis model [9]. Importantly, the treatment could effectively protect bovine endothelial cells from LPS-induced cell death [9]. However, its action in modulating the inflammation response has not been explored yet. Herein, we demonstrate the effect of LPS on the activation of the inflammation response which resulted in cell death in bovine endothelial cells. The anti-inflammation activities of *C. nutans* crude extract and its solvent fractions were tested to lower the upregulation of proinflammatory cytokines (*IL6* and *IL1β*) and chemokines (*CXCL3* and *CXCL8*) and anti-cell death activity after LPS induction. The bioactive compound glyceryl 1,3-distearate (C_39_H_76_O_5_) was determined by thin-layer chromatography (TLC) and tested for its contribution to anti-inflammation and anti-cell death activity. The findings from this study strongly support the use of *C. nutans* extract as an alternative treatment for bovine mastitis.

## 2. Results

### 2.1. LPS-Induced Cell Death and Inflammation in Bovine Endothelial Cells

**Figure 1:**
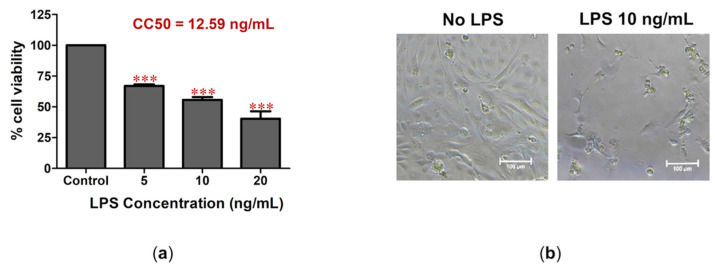
The effect of LPS on CPEA cell death. (**a**) CPAE cell viability was measured after treatment with LPS for 24 h. The percentage of cell viability was analyzed relative to the non-treatment control. The CC50 value was analyzed using non-linear regression with GraphPad Prism Version 5.0. (**b**) The cell morphology of treated cells was monitored under the microscope (*** *p* < 0.001). LPS treatment caused the activation of inflammation. Real-time PCR showed the upregulation of genes involved in acute inflammation (*IL1β* and *IL6*) and the accumulation of immune cells to the site of infection (*CXCL3* and *CXCL8*) upon LPS treatment (Figure 2). Treatment with the highest tested concentration (20 ng/mL) for 24 h significantly caused the upregulation of *IL1β*, *IL6*, *CXCL3*, and *CXCL8* by 2530.3, 37.9, 26.7, and 13.2 folds, respectively, relative to the non-treatment control. The results reflect the role of LPS in inducing inflammation in bovine endothelial cells.

We investigated the effect of LPS derived from *E. coli* to induce the inflammation response and cell death in a bovine endothelial CPAE cell line. Treatment of the cells with low concentrations of LPS at 5, 10, and 20 ng/mL caused strong toxicity to the CPAE cells by lowering cell viability to 67.0%, 55.6%, and 40.3%, respectively (Figure 1a). The half-maximal cytotoxic concentration (CC50) was analyzed using non-linear regression, which showed an LPS CC50 of 12.59 ng/mL in CPAE cells (Figure 1a) and caused a dramatic change in CPAE cell morphology (Figure 1b).

**Figure 2:**
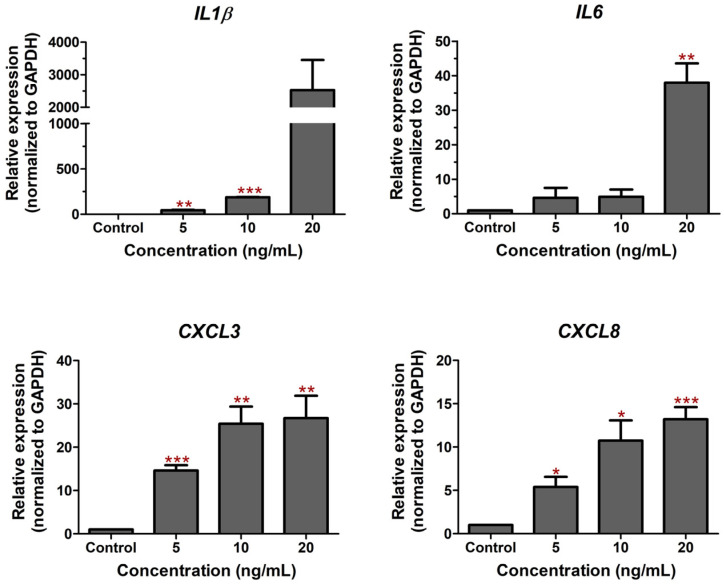
The effect of LPS on the modulation of inflammation-related gene expression. The changes in the expression of pro-inflammatory cytokine genes (*IL1β* and *IL6*) and chemokine genes (*CXCL3* and *CXCL8*) were determined using real-time PCR after treatment with LPS at concentrations of 5, 10, and 20 ng/mL for 24 h in CPAE cells. The gene expression levels were evaluated and normalized with GAPDH (housekeeping genes) and compared with normal cells (without LPS, set as 1.0). (Statistical analysis: * *p* < 0.05, ** *p* < 0.01, and *** *p* < 0.001.)

### 2.2. C. nutans Extract Protected against Cell Death and Lowered Inflammation Response

**Figure 3:**
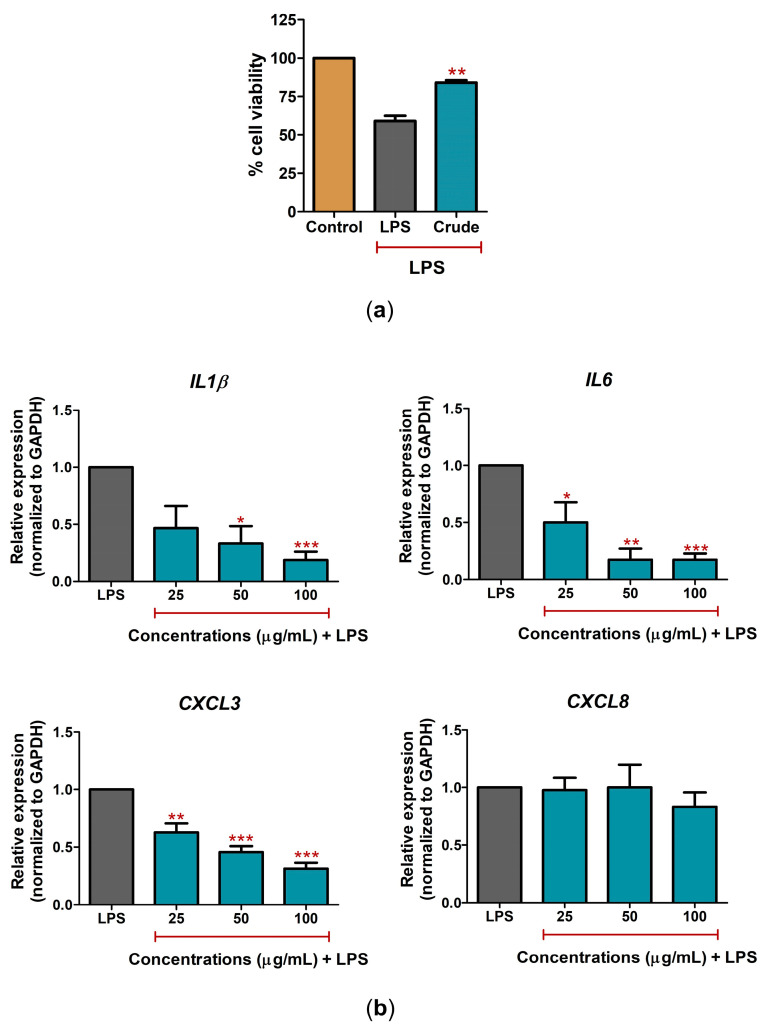
The effect of *C. nutans* extract on rescue from LPS-induced cell death and inflammation in CPEA cells. (**a**) The cell viability after treatment with LPS (10 ng/mL) in the presence or absence of *C. nutans* extract (100 µg/mL) at 24 h. (**b**) The changes in gene expression of *IL1β*, *IL6*, *CXCL3*, and *CXCL8* after LPS treatment (10 ng/mL) with or without *C. nutans* extract (25, 50, and 100 µg/mL) at 24 h as determined by real-time PCR. The gene expression levels were evaluated and normalized with GAPDH (housekeeping genes) and compared with LPS without the *C. nutans* extract (set as 1.0). (Statistical analysis: * *p* < 0.05, ** *p* < 0.01, and *** *p* < 0.001.)

The cytotoxicity test of *C. nutans* extract revealed that the extract at concentrations up to 500 μg/mL had no cytotoxicity effect against CPAE cells (Supplementary Figure S1). Treatment with a non-toxic concentration of *C. nutans* extract (100 μg/mL) could significantly rescue up to 84.0% of cell viability after LPS activation compared to LPS-treated cells without *C. nutans* extract, which caused a reduction in cell viability of 59.0% (Figure 3a). The anti-inflammation activity of *C. nutans* extract was investigated to lower the upregulation of *IL1β*, *IL6*, *CXCL3*, and *CXCL8* expression upon LPS treatment. The results showed that treatment with 25, 50, and 100 μg/mL of *C. nutans* extract could significantly lower *IL1β*, *IL6*, and *CXCL3* expression in a dose-dependent manner, but not the expression of *CXCL8*. At the highest concentration (100 μg/mL), *C. nutans* extract decreased the gene expression of *IL1β*, *IL6*, and *CXCL3* in LPS-treated cells by 0.19, 0.17, and 0.31 folds, respectively, relative to that of the LPS-treated cells without the extract (set as 1.0) (Figure 3b). However, the treatment caused only a slight change, reducing *CXCL8* expression 0.83-fold (Figure 3b).

### 2.3. C. nutans Extract Fractions Exerted Anti-Cell-Death and Anti-Inflammation Activity

**Figure 4:**
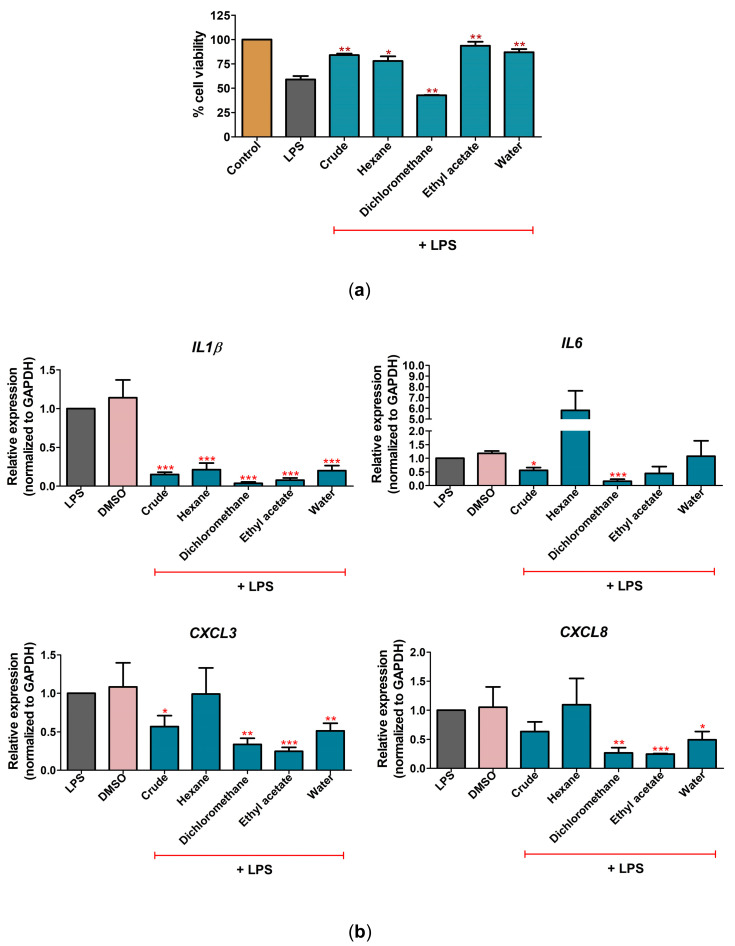
The effect of *C. nutans* extract fractions on rescue from LPS-induced cell death and inflammation in CPEA cells. (**a**) The cell viability after treatment with LPS (10 ng/mL) in the presence or absence of *C. nutans* extract fractions (100 μg/mL) at 24 h. (**b**) The changes in gene expression of *IL1β*, *IL6*, *CXCL3*, and *CXCL8* after LPS treatment with or without *C. nutans* extract fractions at 24 h as determined by real-time PCR. The gene expression levels were evaluated and normalized with GAPDH (housekeeping genes) and compared with LPS without the *C. nutans* extract fractions (set as 1.0). (Statistical analysis: * *p* < 0.05, ** *p* < 0.01, and *** *p* < 0.001.)

The fractionation of *C. nutans* extract was performed by sequential extraction of hexane (C_6_H_14_), dichloromethane (CH_2_Cl_2_), ethyl acetate (C_4_H_8_O_2_), and water (H_2_O). All fractions were determined for their biological activities in reducing cell death and lowering mRNA expression of *IL1β*, *IL6*, *CXCL3*, and *CXCL8* in LPS-treated CPAE cells. At equal concentrations (100 μg/mL), *C. nutans* fractions, except for the dichloromethane fraction, significantly rescued cell viability in LPS-treated cells (Figure 4a). The cytotoxicity of all fractions was tested, and the results showed that only the dichloromethane fraction significantly caused a reduction in cell viability (Supplementary Figure S2), which suggested that the lower activity of the dichloromethane fraction in rescue from LPS-induced cell death might have been due to its cytotoxicity to the cells. Interestingly, treatment with *C. nutans* fractions, except for the hexane fraction, potentially lowered the upregulation of *IL1β*, *IL6*, *CXCL3*, and *CXCL8*. The ethyl acetate fraction was the most effective fraction in protecting 93.6% of cell viability from LPS (Figure 4a), in addition to lowering the inflammation-related gene expression of *IL1β*, *IL6*, *CXCL3*, and *CXCL8* by 0.08, 0.45, 0.25, and 0.25 folds, respectively, relative to the LPS-treated cells without the *C. nutans* extract (set as 1.0) (Figure 4b).

### 2.4. The Bioactive Compound Glyceryl 1,3-Distearate Promoted Anti-Inflammation Activity 

The bioactive compound glyceryl 1,3-distearate (C_39_H_76_O_5_) has been reported as the major compound in *C. nutans* extracts previously [9]. We confirmed the presence of glyceryl 1,3-distearate in *C. nutans* fractions in which hexane (C_6_H_14_), dichloromethane (CH_2_Cl_2_), ethyl acetate (C_4_H_8_O_2_), and water (H_2_O) were used as solvents in TLC analysis. The results revealed that glyceryl 1,3-distearate was found in all fractions with a retention factor of 0.48 (Supplementary Figure S3). According to the chemical properties of glyceryl 1,3-distearate, which is comprised of long-chain fatty acids, its solubility capacity would be different in the different organic solvents depending on the polarity. Based on our TLC results, this compound was found in all *C. nutans* fractions, suggesting that this compound could be soluble in polar and non-polar solvents; however, the amount of glyceryl 1,3-distearate in these fractions could not be assessed by the TLC method.

Furthermore, GC-MS/MS analysis was performed to demonstrate the presence of glyceryl 1,3-distearate and explore the possible other bioactive compounds. The extract contained various compounds and metabolites, mainly fatty acid, glycoside, terpenoid, cyclopentane, and disulfide groups (Table 1). Glyceryl 1,3-distearate was identified as one of the fatty acids, which clearly confirmed our previous report that glyceryl 1,3-distearate was the major bioactive compound in *C. nutans* extract based on LC-MS/MS analysis [9]. 

**Figure 5:**
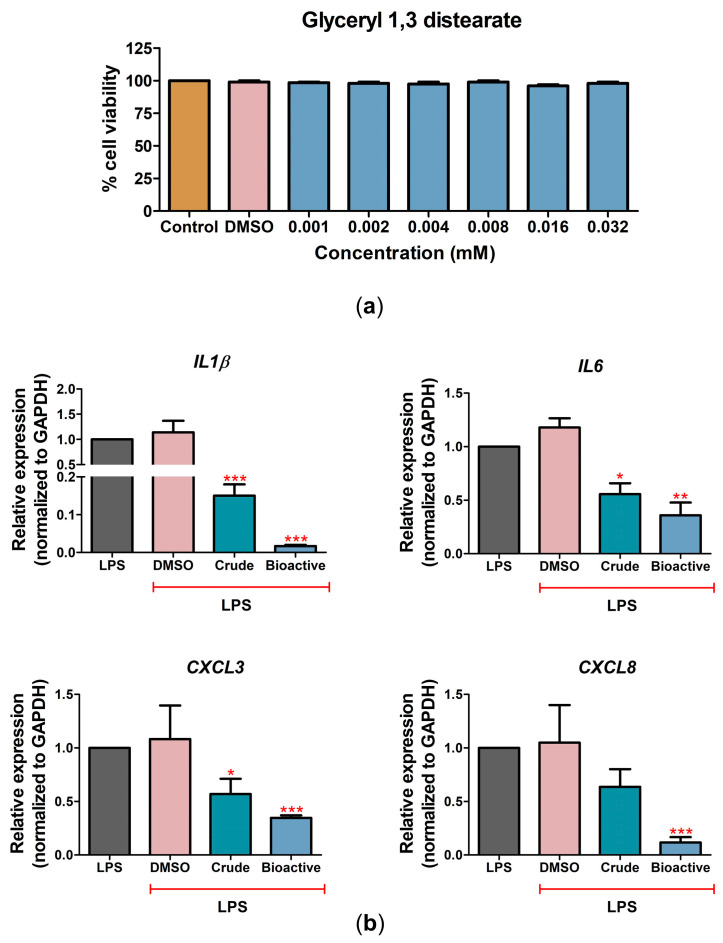
The effects of glyceryl 1,3-distearate (bioactive compound) in CPAE cells. (**a**) The cell viability after treatment with glyceryl 1,3-distearate (0.001–0.032 mM, two-fold dilution) at 24 h. (**b**) The changes in gene expression of *IL1β*, *IL6*, *CXCL3*, and *CXCL8* after treatment with LPS combined with *C. nutans* extract or glyceryl 1,3-distearate at 24 h as determined by real-time PCR. The gene expression levels were evaluated and normalized with GAPDH (housekeeping genes) and compared with LPS without the *C. nutans* extract (set as 1.0). (Statistical analysis: * *p* < 0.05, ** *p* < 0.01, and *** *p* < 0.001.)

Cytotoxicity was investigated to evaluate the effect of glyceryl 1,3-distearate on CPAE cell viability. Glyceryl 1,3-distearate concentrations up to 0.032 mM had no cytotoxicity effects according to a cell viability assay (Figure 5a). Treatment with various concentrations of glyceryl 1,3-distearate (0.001–0.016 mM) tended to decrease cell death after LPS treatment. Glyceryl 1,3-distearate at concentrations of 0.008 and 0.016 mM could rescue 74.6% and 80.3% of cell viability, respectively, compared with LPS-treated cells without treatment with the bioactive compound (Supplementary Figure S4). Interestingly, the crude extract or glyceryl 1,3-distearate treatment showed anti-inflammation activity to lower the expression of inflammatory genes (Figure 5b). Compared to the crude extract (100 μg/mL), glyceryl 1,3-distearate (0.032 mM) had more efficacy in lowering the expression levels of *IL1β* 0.02-fold, *IL6* 0.36-fold, *CXCL3* 0.35-fold, and *CXCL8* 0.12-fold, relative to LPS-treated cells without *C. nutans* extract (set as 1.0). The results suggested the contribution of glyceryl 1,3-distearate as an anti-inflammation compound in lowering the magnitude of inflammation in LPS-treated bovine endothelial cells (Figure 5b).

## 3. Discussion

Plant-based drugs have been widely used in traditional medicine in several countries for many decades and they are well-recognized for their lower toxicities and great potential in treating drug-resistant disease [4], pointing to the effectiveness of natural products as being greatly aligned with modern medicine. Herbal medicine remains the major healthcare [18] system for around 80% of the world’s population, particularly in developing countries, and provides complementary and alternative medicines in the line of primary healthcare. Apart from traditional uses, the scientific evidence supporting therapeutic activities, benefits in treating certain diseases, and safety evaluations could be critical in elevating the acceptance of medicinal plants for application in mainstream therapeutics. Several studies have demonstrated the diverse effects of *C. nutans*, suggesting the potential of *C. nutans* in the development of alternative plant-based drugs [2,4,19]. However, the therapeutic use of *C. nutans* extract available in the market is limited to a few diseases. To expand the use of *C. nutans* extract as a complementary therapeutic option, we investigated the therapeutic activity of *C. nutans* extract in bovine mastitis, which is the most significant bacterial infectious disease affecting the dairy industry worldwide.

Bacterial infection can lead to the injury and eventually result in the death of cells through apoptotic pathways, particularly at the site of infection in mammary glands [20]. One of the most sensitive cell types to bacterial infection is endothelial cells, based on the literature, which has demonstrated LPS as a serious cause of uncontrolled inflammation responses and endothelial cell integrity loss [21]. Previously, we reported the anti-cell death activity of *C. nutans* extract against LPS induction in bovine endothelial cells in what was the first report to uncover the benefit of *C. nutans* in bovine mastitis treatment [9]. In the present study, we confirmed the effect of LPS in inducing cell death (Figure 1) and that treatment with *C. nutans* extract could potentially inhibit this consequence by lowering the number of dead cells (Figure 3a).

Indeed, cell death is the ultimate consequence of bacterial infection, the intermediated response, including the inflammation response, acting as the main driver in promoting cell death. The response to LPS is a key factor in the pathogenicity of gram-negative bacteria that promote pathological effects in host cells. In intramammary infections, pathogen-associated molecular patterns (PAMPs), including LPS, are recognized by Toll-like receptors (TLRs) of host cells. These interactions promote the induction of inflammation cascades to release immune-modulating cytokines. Our study demonstrated the effect of LPS in upregulating the expression of inflammatory cytokines and chemokines, including *IL1β*, *IL6*, *CXCL8 (IL8)*, and *CXCL3* (Figure 2), which have been reported to be involved in LPS response previously [15]. Interestingly, treatment with *C. nutans* extract and its solvent fractions could significantly decrease these expressions after LPS stimulation (Figure 3b and Figure 4b), in accordance with a report that recently revealed the effect of *C. nutans* extract in reducing inflammation in brain endothelial cells after treatment with high-dose 7-ketocholesterol (cholesterol oxidation product) [22]. The LPS treatment extremely increased the expression of the pro-inflammatory cytokines *IL1β*, *IL6*, *CXCL8*, and *TNF-α*, but *C. nutans* extract treatment could diminish these upregulations significantly, which emphasizes the potential for *C. nutans* extract as an anti-inflammation agent.

We previously identified the major bioactive compound in *C. nutans* solvent fractions as glyceryl 1,3-distearate. Herein, we confirmed the presence of glyceryl 1,3-distearate in the crude extract and determined its ability to lower cell death and inflammation (Figure 5 and Figure S3). Treatment with glyceryl 1,3-distearate could significantly diminish the upregulation of inflammatory genes, namely, *IL1β*, *IL6*, *CXCL8*, and *CXCL3* (Figure 5b); however, it had less effect in protecting against LPS-induced cell death compared to the crude extract (Supplementary Figure S4). In addition, glyceryl 1,3-distearate at the highest tested concentration (1.6 mM) had no antibacterial activity according to a disc agar diffusion assay (Supplementary Table S1). These results suggest the existence of other bioactive compounds apart from glyceryl 1,3-distearate in the crude extract which contribute to the anti-apoptosis and anti-bacterial activities of *C. nutans* extract. Notably, the crude extract provided a broad-range activity as a result of the natural combination of bioactive compounds. The bioactive compounds in the crude extract had a high possibility for a synergistic effect through the different intracellular targets or different signaling pathways which amplified and provided the improvement of therapeutic action. Likewise, our finding is a proof of concept for the potential beneficial use of *C. nutans* crude extract, since it exerts greater broad-range biological activity than the single bioactive compound.

Apart from biological activity, safety is a critical issue in developing herb extracts as alternatives treatments. As discussed above, *C. nutans* extract has been used in traditional medicine for humans; however, in vivo cytotoxicity studies in animal models are still required due to the lack of information on its use in cows. Although our experiment suggested that *C. nutans* extract was highly safe concentrations up to 0.5 mg/mL caused no effect on cell viability further investigation in cows is important to assess the safety and therapeutic benefit of *C. nutans* extract. In conclusion, our findings revealed the potential of *C. nutans* extract to be used as an alternative therapy to minimize LPS effects on cell death and inflammation.

## 4. Materials and Methods

### 4.1. Herb Extracts

*C. nutans* plants were purchased at the organic market at Chiang Mai University, Thailand, in 2019. Dr. Narin Printarakul, a taxonomist from the Department of Biology, Faculty of Science, Chiang Mai University, identified and produced voucher specimen no. CN001-003. The leaves of the *C. nutans* were cleaned and dried at 50 °C in a hot-air oven. The plants were ground to powder form using a grinder. The powder of the herb was extracted using 70% ethanol (the herbs were mixed with 70% ethanol at a 1:20 ratio). The samples were shaken at 160 rpm/min 25 °C for 12 h, followed by filtration using Whatman No. 1 filter papers. After that, the extracts were evaporated in a rotary evaporator. The residues were dried thoroughly to remove the solvents used in a water bath at 95 °C. The weights were calculated for the % yield of the extract, and the samples were stored at 4 °C until use, when the extracts were dissolved using DMSO for treated cells.

% yield of the extract = [(weight of the dry extracts/weight of the dry plant) × 100]

### 4.2. Fractionation of Herb Extracts

The 70% ethanolic crude extracts of *C. nutans* were solubilized with 5% MeOH/water (*v*/*v*). The fractionated crude extracts were separated into 4 fractions in order of their polarities: a hexane fraction (C_6_H_14_), a dichloromethane fraction (CH_2_CI_2_), an ethyl acetate fraction (C_4_H_8_O_2_), and an aqueous fraction (H_2_O). Briefly, hexane was used to partition three times with MeOH part at a 1:1 (*v*/*v*) ratio. After that, the methanol part was divided further using a 1:1 (*v*/*v*) ratio of dichloromethane, followed by ethyl acetate at a 1:1 (*v*/*v*) ratio. After that, the extract fractions were evaporated using a rotary evaporator, dried in a laboratory fume hood, and stored at 4 °C until use, when the extracts were dissolved using DMSO for treated cells.

### 4.3. Cell Lines and Reagents

The bovine endothelial cell line, namely, CPAE (CCL209TM) (ATCC, Manassas, VA, USA), was cultured in the minimal essential medium (MEM). The medium was supplemented with 20% (*v*/*v*) fetal bovine serum (FBS) and antibiotics (penicillin G and streptomycin) at 37 °C in a 5% CO_2_ humidified atmosphere.

### 4.4. Cell Viability

Cell viability was determined after LPS treatment (L4391-1MG, *E. coli* 0111: B4, Sigma-Aldrich, St. Louis, MO, USA) in the CPAE cells. The cells were plated a day before the experiment in 96-well plates (7000 cells/well) and treated with 5, 10, and 20 ng/mL of LPS. To investigate the effects of the *C. nutans* crude extracts and glyceryl 1,3-distearate (Sigma-Aldrich, St. Louis, MO, USA) in lowering the effect of LPS on cell death, cell viability was measured after LPS treatment (10 ng/mL) in the presence of *C. nutans* crude extracts (100 μg/mL) and glyceryl 1,3-distearate (0.001, 0.002, 0.004, 0.008, 0.016, and 0.032 mM). Twenty-four hours after treatment, the cell viability was determined using PrestoBLUE™ cell viability reagent (Thermo Fisher Scientific, Waltham, MA, USA). The absorbance variations in the color of the reagent were measured at 570 nm and 595 nm using a microplate reader (EZ Read 2000, Biochrom, Cambridge, UK). The data were analyzed to calculate the percentage of cell viability relative to that of the non-treated control using the following equation.

% cell viability = [(OD570-OD595) treated cells/(OD570-OD595) non-treated cells] × 100

The half-maximal cytotoxicity concentration (CC50) was analyzed using regression analysis (GraphPad Prism version 5.0, San Diego, CA, USA).

### 4.5. Real-Time PCR

The effects of the *C. nutans* crude extracts and glyceryl 1,3-distearate on the expression of inflammatory genes were determined in LPS-treated CPEA cells. Briefly, the cells were plated the day before the experiment in 12-well plates (50,000 cells/well). The cells were treated with LPS (10 ng/mL) in the absence or presence of *C. nutans* crude extracts (25, 50, and 100 μg/mL), *C. nutans* fractions (equal concentrations = 100 μg/mL), or glyceryl 1,3-distearate (0.032 mM). At 24 h of incubation, the RNA was extracted from the treated cells using TRIzol^®^ reagent (Invitrogen, Carlsbad, CA, USA). The purity and integrity of RNA were verified (OD260/OD280 ratio > 1.8). The RNA was used as the template to synthesize cDNA by using ReverTra Ace^®^ qPCR RT Master Mix (Toyobo Life Science, Osaka, Japan). The real-time PCR reactions were performed using SensiFAST™ SYBR^®^ No-ROX Kit (Bioline, London, UK) with specific primers (Table S2, Supplementary Materials). Primer pairs for all genes were designed and analyzed using DNA sequences from the NCBI database “http://www.ncbi.nlm.nih.gov/ (accessed on 20 June 2020)”. Gene expression was analyzed as fold changes via the 2^−∆∆CT^ method, and the housekeeping gene *GADPH* was used to normalize the gene expression.

### 4.6. Thin-Layer Chromatography (TLC) Analysis

*C. nutans* crude extracts, each fraction of the *C. nutans* extracts, and C_39_H_76_O_5_ were spotted on the TLC plate. Then, TLC silica gel plate was developed in the mobile phase of hexane/chloroform/ethyl acetate/methanol/water/formic acid (3:3:2:2:0.1:0.1). The developed TLC plate was visualized by spraying with 10% H_2_SO_4_ reagent at 120 °C for 5 min. The TLC plate was visualized under UV light at 366 nm. The retention factor (Rf) was calculated as follows:

Rf = distance traveled by the compound/distance traveled by the solvent front

### 4.7. Gas Chromatography-Mass Spectrometry/Mass Spectrometry (GC-MS/MS) Analysis

The phytochemical analysis of the *C. nutans* crude extracts was performed using GC-MS/MS equipment (GC 7890B Agilent Technology, CA, USA) with a DB-5MS Agilent fused silica capillary column (30 m × 0.25 mm ID; film thickness: 0.25 µm). The experimental condition for the GC-MS system was as follows: the flow rate of the mobile phase (carrier gas: He) was set at 1.0 mL/min. The sample was dissolved in ethyl acetate solution and filtrated through a 0.22 µm nylon membrane filter before injection. The 1 µL of the sample was injected (split ratio 10:1). The column oven temperature was set at 50 °C for 2 min, raised up to 5 °C/per min up to 230 °C, held for 1 min, then raised up again 5 °C/per min up to 280 °C, held for 2 min, and the final temperature was increased up to 300 °C for 5 min. The phytochemicals were identified by a comparison of their retention times (min), peak areas, and mass spectra with the spectral data of authentic compounds stored in the National Institute of Standards and Technology (NIST17) libraries.

### 4.8. Statistical Analysis

The data from at least three independent experiments were used to analyze the statistical differences using the student’s *t*-test (GraphPad Prism version 5.0, San Diego, CA, USA): * indicates *p* < 0.05, ** indicates *p* < 0.01, and *** indicates *p* < 0.001.

## 5. Conclusions

Our results indicate that *C. nutans* extracts and glyceryl 1,3-distearate have the ability to reduce the mRNA expression of *IL1β*, *IL6*, *CXCL3*, and *CXCL8* in LPS-treated cells. *C. nutans* extracts have no toxicity in CPEA cells and could be used as medicines in defense against bacterial infection and bovine mastitis. However, further studies, especially clinical tests, should be carried out in the future.

## Figures and Tables

**Table 1:** The phytochemical composition of ethanolic leaf extract of *C. nutans* as determined by GC-MS/MS analysis.

No.	Classification	IUPAC Name	Name of Compound	Molecular Formula	Peak Area	RT
1	Fatty Acids	1-ethoxybutane	Butane, 1-ethoxy-	C_6_H_14_O	185,779 (15)	3.496
2		propyl acetate	n-Propyl acetate	C_5_H_10_O_2_	3,357,413 (04)	3.733
3		butan-2-yl acetate	sec-Butyl acetate	C_6_H_12_O_2_	1,647,493 (07)	4.465
4		1-butoxybutane	n-Butyl ether	C_8_H_18_O	267,510 (14)	7.555
5		2-ethylhexyl hexyl sulfite	Sulfurous acid	C_14_H_30_O_3_S	168,824 (16)	20.671
6		octadecanoic acid	Hexadecanoic acid	C_18_H_36_O_2_	2,304,434 (05)	36.235
7		methyl (11E,14E,17E)-icosa-11,14,17-trienoate	11,14,17-Eicosatrienoic acid	C_21_H_36_O_2_	1,199,156 (10)	38.983
8		ethyl (9Z,12Z,15Z)-octadeca-9,12,15-trienoate	9,12,15-Octadecatrienoic acid	C_20_H_34_O_2_	4,601,038 (02)	39.635
9		ethyl octadecanoate	Octadecanoic acid	C_20_H_40_O_2_	1,615,070 (08)	40.169
10		dioctyl hexanedioate	Hexanedioic acid	C_22_H_42_O_4_	901,322 (12)	43.862
11		2,3-dihydroxypropyl hexadecanoate	Hexadecanoic acid	C_19_H_38_O_4_	1,532,912 (09)	45.993
12		(2-hydroxy-3-octadecanoyloxypropyl) octadecanoate	Glyceryl 1,3-distearate	C_39_H_76_O_5_	1,954,834 (06)	49.320
13	Glycosides	(2S,3R,4S,5S,6R)-2-ethoxy-6-(hydroxymethyl) oxane-3,4,5-triol	Ethyl alpha-d glucopyranoside	C_8_H_16_O_6_	949,858 (11)	28.386
14	Terpenoids	(E,7R,11R)-3,7,11,15-tetramethylhexadec-2-en-1-ol	Phytol	C_20_H_40_O	4,125,560 (03)	38.460
15	Cyclopentanes	cyclopent-4-ene-1,3-dione	4-Cyclopentene-1,3-dione	C_5_H_4_O_2_	503,713 (13)	7.986
16	Disulfides	(methyldisulfanyl)methane	Dimethyl disulfide	C_2_H_6_S_2_	5,088,889 (01)	4.298

## Data Availability

Not applicable.

## Supplementary Materials

The following supporting information can be downloaded at: https://www.mdpi.com/article/10.3390/antibiotics12030549/s1, Figure S1: The cytotoxicity effect of *C. nutans* extract on CPAE cells; Figure S2: The cytotoxicity effects of *C. nutans* fractions on CPAE cells; Figure S3: TLC chromatogram of *C. nutans* fractions; Figure S4: The cytotoxicity effect of glyceryl 1,3-distearate on CPAE cells treated with LPS; Table S1: Antibacterial activity of glyceryl 1,3-distearate against *E. coli* using the agar disc diffusion method; Table S2: List of real-time PCR primers.
