# Supplementary material for: Anti-Inflammatory Activity of Glyceryl 1,3-Distearate Identified from Clinacanthus nutans Extract against Bovine Mastitis Pathogens
Source: Antibiotics (Basel). 2023 Mar 9;12(3):549. doi: 10.3390/antibiotics12030549 (PMC10044565; doi:10.3390/antibiotics12030549)
Supplement: Supplementary file 1 [file antibiotics-12-00549-s001.zip › antibiotics-2200755-supplementary.pdf]

Supplementary data

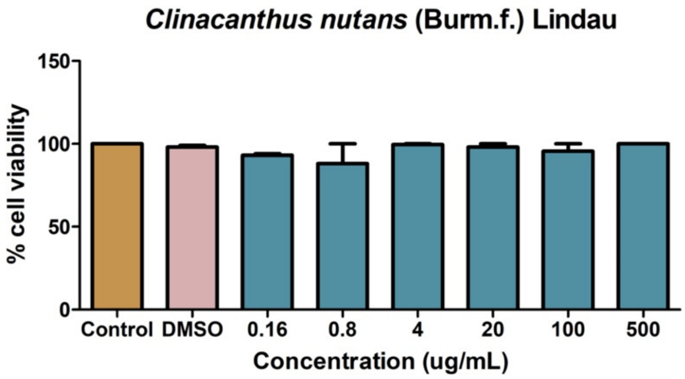

Figure S1 The cytotoxicity effect of *C. nutans* extract on CPAE cells. The cells were plated the day prior to the experiment at a density of approximately 7000 cells/well in the 96-well plates. At the time of experiment, the crude extract was prepared in serial concentrations ranged from 0.16-500 µg/mL and added to the CPAE cells (100 µL per well). After 24 hours of treatment, the cells were investigated for the cell viability by using PrestoBLUE™ cell viability reagent. The data was analyzed as the percentage of cell viability where the non-treatment control was set as 100%.

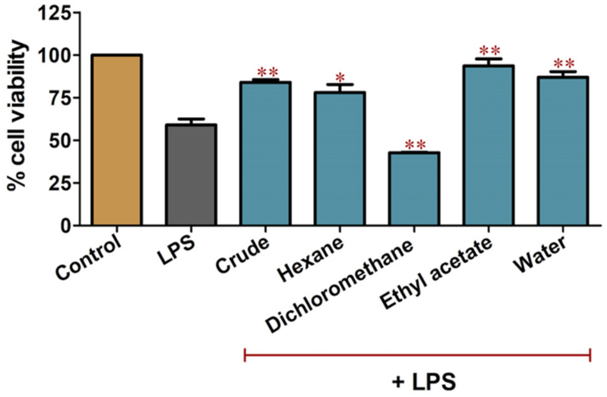

Figure S2 The effect of C. nutans extract fractions to rescue the LPS-induced cell death. The cells were plated the day prior to the experiment at a density of approximately 7000 cells/well in the 96-well plates. The cell viability after treatment with LPS (10 ng/mL) in the presence or absence of *C. nutans* extract fractions (100 µg/mL) at 24 hours. After 24 hours of treatment, the cells were investigated for the cell viability by using PrestoBLUE™ cell viability reagent. The data was analyzed as the percentage of cell viability where the non-treatment control was set as 100%.

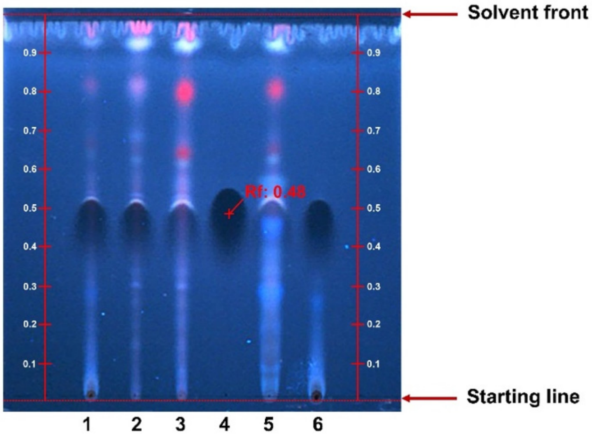

Figure S3 TLC chromatogram of *C. nutans* fractions (1); hexane fraction (2); dichloromethane fraction (3); glyceryl 1,3 distearate, Rf 0.48 (4); ethyl acetate fraction (5); and water fraction (6) at UV 366 nm.

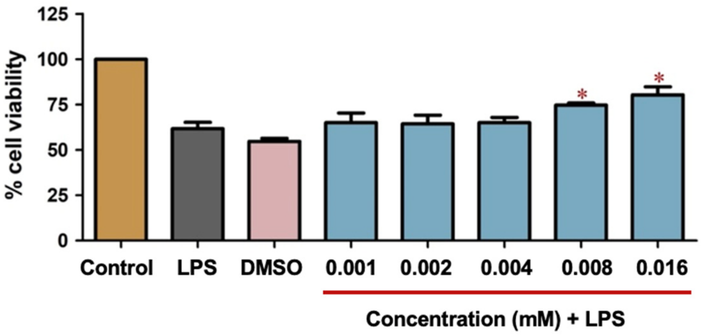

Figure S4 The effect of glyceryl 1,3 distearate to rescue the LPS-induced cell death and inflammation in CPEA cells. The cells were plated the day prior to the experiment at a density of approximately 7000 cells/well in the 96-well plates. At the time of experiment, LPS (10 ng/mL) was treated to the cells in presence or absence of glyceryl 1,3 disterate was prepared in serial concentrations ranged from 0.001-0.016 mM and added to the CPAE cells (100 µL per well). After 24 hours of treatment, the cells were investigated for the cell viability by using PrestoBLUE™ cell viability reagent. The data was analyzed as the percentage of cell viability where the non-treatment control was set as 100%.

Table S1 Antibacterial activity of glyceryl 1,3-disterate against *E. coli* using agar disc diffusion method. The clear zone was measured and summarized in the table (mean ± standard deviation) where the gentamycin was used as the positive control.

Herb Extract	Clear Zone (mm)
Clinacanthus nutans (Burm.f.) Lindau (500 mg/mL)	7 ± 0.00
Glyceryl 1,3-disterate (1.6 mM)	0.00
Gentamycin (2 mM)	20.33 ± 1.52

Table S2 List of real-time PCR primers.

Genes	Forward primer (5'→3')	Reverse primer (5'→3')
IL1β	GAGGCTGATGGCCCTAAACA	GTAGGCACTGTTCCTCAGCTT
IL6	CACCCCAGGCAGACTACTTC	CCCAGATTGGAAGCATCCGT
CXCL3	ATACAGAGCGTGAAGGTGACG	ATGGGAGCTTCAGGGTTGAG
CXCL8	ATTCCACACCTTTCCACCCC	ACCCACTTTTCCTTGGGGTT
GADPH	GCTGCCCAGAATATCATCCCT	GCAGGTCAGATCCACAACAG
